# Mitochondria-Targeted Nitronyl Nitroxide Radical Nanoparticles for Protection against Radiation-Induced Damage with Antioxidant Effects

**DOI:** 10.3390/cancers16020351

**Published:** 2024-01-13

**Authors:** Shigao Huang, Min Xu, Qingyue Da, Linlin Jing, Haibo Wang

**Affiliations:** 1 Department of Cell Biology, National Translational Science Center for Molecular Medicine, The Air Force Medical University, Xi’an 710032, China; 2 Department of Radiation Oncology, Xijing Hospital, The Air Force Medical University, Xi’an 710032, China; 3 Department of Chemistry, School of Pharmacy, The Air Force Medical University, Xi’an 710032, China; 4 Centre for Translational Medicine, The First Affiliated Hospital, Xi’an Jiaotong University, Xi’an 710061, China; daqy21@lzu.edu.cn (Q.D.); jinglinlin@xjtufh.edu.cn (L.J.)

**Keywords:** ionizing radiation, nitronyl nitroxide radical, radioprotective effect, mitochondria-targeted, tumor radiotherapy

## Abstract

**Simple Summary:**

Radiation therapy is widely applied to treat and alleviate cancers. However, it may lead to acute and chronic side effects in adjacent normal organs. Therefore, it is imperative to develop effective strategies for the radiological protection of the normal organs and immune system of patients during radiotherapy. In this context, we investigated a mitochondria-targeted nitronyl nitroxide radical with a tri-phenylphosphine ion (TPP-NIT) and its nanoparticles (NPs-TPP-NIT) with minimal toxicity. Through in vitro and in vivo experimental studies, we found that NPs-TPP-NIT display a protective effect against oxidative damage caused by X-ray irradiation. These findings reveal that NPs-TPP-NIT may be promising radioprotectors and could therefore be applied to protect healthy tissues and organs from radiation during the treatment of cancer with radiotherapy.

**Abstract:**

Radiotherapy is a non-invasive method that is widely applied to treat and alleviate cancers. However, radiation-induced effects in the immune system are associated with several side effects via an increase in oxidative stress and the inflammatory response. Therefore, it is imperative to develop effective clinical radiological protection strategies for the radiological protection of the normal organs and immune system in these patients. To explore more effective radioprotective agents with minimal toxicity, a mitochondria-targeted nitronyl nitroxide radical with a triphenylphosphine ion (TPP-NIT) was synthesized and its nanoparticles (NPs-TPP-NIT) were prepared and characterized. The TPP-NIT nanoparticles (NPs-TPP-NIT) were narrow in their size distribution and uniformly distributed; they showed good drug encapsulation efficiency and a low hemolysis rate (<3%). The protective effect of NPs-TPP-NIT against X-ray irradiation-induced oxidative damage was measured in vitro and in vivo. The results show that NPs-TPP-NIT were associated with no obvious cytotoxicity to L-02 cells when the concentration was below 1.5 × 10^−2^ mmol. NPs-TPP-NIT enhanced the survival rate of L-02 cells significantly under 2, 4, 6, and 8 Gy X-ray radiation exposure; the survival rate of mice was highest after 6 Gy X-ray irradiation. The results also show that NPs-TPP-NIT could increase superoxide dismutase (SOD) activity and decrease malondialdehyde (MDA) levels after the L-02 cells were exposed to 6.0 Gy of X-ray radiation. Moreover, NPs-TPP-NIT could significantly inhibit cell apoptosis. NPs-TPP-NIT significantly increased the mouse survival rate after irradiation. NPs-TPP-NIT displayed a marked ability to reduce the irradiation-induced depletion of red blood cells (RBCs), white blood cells (WBCs), and platelets (PLTs). These results demonstrate the feasibility of using NPs-TPP-NIT to provide protection from radiation-induced damage. In conclusion, this study revealed that NPs-TPP-NIT may be promising radioprotectors and could therefore be applied to protect healthy tissues and organs from radiation during the treatment of cancer with radiotherapy.

## 1. Introduction

Cancer is among the leading causes of death worldwide [1,2]. Radiotherapy is one of the traditional and promising areas for patients in the treatment of cancer, mainly due to its non-invasive nature and the low incidence of side effects, as well as its suitability for patients who are resistant to current drug therapies [3,4]. Immunotherapy plus radiotherapy is also developing rapidly [5,6]. However, exposure to ionizing radiation (IR) from radiotherapy is a major concern due to the resultant adverse effects on people’s tissues and functions induced by IR [7,8]. Damage induced by ionizing radiation can be caused in two ways. One is direct injury from high-energy radiation, which can cause damage to various biological macromolecules, such as DNA, proteins, and mitochondria. The other type is indirect damage resulting from water ionization, leading to a series of free radical chain reactions, producing various free radicals, such as hydrogen peroxide (H_2_O_2_), hydroxyl radicals (∙OH), and superoxide anions (O_2_^•^) [9]. Reactive oxygen species (ROS) can act on the mitochondrial membrane potential, cause changes in cellular physiology and biochemistry, and induce cell apoptosis. The complex reactions of biomolecules caused by these processes lead to widespread damage to normal tissues and functions. 

Radiation therapy cures patients, reduces physical pain, and prolongs patient lifespans by applying high-energy radiation to tumors to shrink or remove them and/or to prevent cancer recurrence. However, this treatment method is not specific to cancer cells, which may lead to acute and chronic side effects in adjacent normal organs, including intestinal inflammation, bleeding or diarrhea, tissue fibrosis, secondary cancer, and infertility [10]. 

Some endogenous antioxidant enzymes, such as superoxide dismutase, catalase and glutathione peroxidase, have the ability to scavenge ROS and play an important role in protecting mitochondrial function and repairing the DNA damage induced by reactive oxygen species [10]. Recently, exploring radioprotective agents that can effectively protect against radiation damage has attracted widespread attention. So far, some radioactive protective agents in clinical use have been approved by the Food and Drug Administration (FDA), such as amifostine (WR2721, Cumberland Pharmaceuticals Inc., Nashville, TN, USA), Leukine^®^ (sargramostim, Sanofi-Aventis, Paris, France), Neulasta^®^ (pegfilgrastim, Amgen, Thousand Oaks, CA, USA), and Neupogen^®^ (filgratism, Amgen, Thousand Oaks, CA, USA); among these agents, amifostine is the most widely used [11]. However, WR2721 has some serious side effects, including hypotension, vomiting, and nausea. In addition to sulfhydryl compounds, other compounds that protect against radiation, such as vitamins, amines, hormones, biological agents, and traditional Chinese medicine, are also the focus of research [12,13]. Researchers have recently investigated the efficacy of many natural or synthetic/semisynthetic chemicals to reduce the adverse effects of ionizing radiation, due to the inherent toxicity of some synthetic chemicals at the concentration required for protection against radiation. However, it is necessary to develop radioprotective agents with improved activity and less toxic side effects. 

Nitronyl nitroxide radicals (NRs) are stable free radical compounds that have single-spin electrons. NRs are classified into two types: TEMPO (2,2,6,6-tetramethyl piperidine-*N*-oxyl) and nitronyl nitroxyl radicals (NITs). In recent years, it has been proven that NRs are effective free radical scavengers and have a certain protective effect against radiation. NRs can react with ROS produced by catalytically ionizing radiation and prevent biomacromolecules and biofilms from being attacked by ROS to protect the body against oxidative damage [14,15]. 

NPs are widely used in the clinic for therapeutic purposes. Sarkar Siddique et al. reviewed the application of nanomaterials in biomedical imaging and cancer therapy [16,17]. Therapeutic NPs improve the accumulation and release of pharmacologically active agents at the pathological site, which increases therapeutic efficacy and reduces the incidence and intensity of side effects overall. Poly(lactic-co-glycolic-acid) (PLGA), a biodegradable nanodrug carrier with a high degree of biological safety, is formed by the copolymerization of two monomers, poly(lactic acid) and poly(glycolic acid), in different proportions. It has been recognized as a widely used nanodrug carrier and tissue engineering scaffolding material by the FDA and the European Medicines Agency.

**Figure 1:**
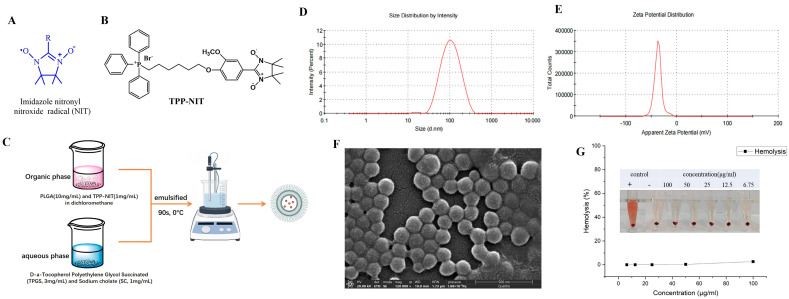
Preparation and the characteristics of NPs-TPP-NIT. (**A**,**B**) The chemical structure of NIT and TPP-NIT. (**C**) The preparation method of the nanoparticles. (**D**) Particle size. (**E**) The zeta potential of TPP-NIT (1 mg/mL) loaded nanoparticles were measured at 25 °C via the DLS technique using Nano ZSP Zetasize. (**F**) Nanoparticles were characterized using scanning electron microscopy at a magnification of 120,000. (**G**) Blood compatibility study of nanoparticles. The concentration was 100 µg/mL, 50 µg/mL, 25 µg/mL, 12.5 µg/mL, and 6.25 µg/mL, and the solution of NP-TPP-NIT was mixed with an equal volume of the erythrocyte suspension. The absorbance of the sample supernatant at 490 nm was measured using spectrophotometry.

Therefore, with the aim of developing agents that protect against IR more effectively and with less toxicity, we synthesized a new NIT radical derivative with a triphenylphosphine ion (TPP-NIT; Figure 1B). Furthermore, we used PLGA as a nanocarrier to encapsulate the mitochondrial-targeting NIT radical to form nanoparticles (NPs-TPP-NIT; Figure 1C). Furthermore, we studied the suppressive effect of NPs-TPP-NIT on the oxidative stress damage in vitro and in vivo. The role of nitronyl nitroxide radical nanoparticles (NPs-TPP-NIT) in the protection of normal cells and healthy tissues from irradiation was studied [18]. Therefore, in this study, we developed a protocol to apply NPs-TPP-NIT as radiotherapy in order to reduce the side effects of X-radiation on healthy tissues and organs.

## 2. Materials and Methods

### 2.1. Preparation and Characterization of NPs-TPP-NIT

#### 2.1.1. Chemical Materials 

The (4-hydroxy-3-methoxyphenyl)-4,4,5,5-tetramethyl-4,5-dihydro imidazole radical (TPP-NIT) was synthesized in our laboratory [19]. Amifostine (WR2721) was purchased from MedChemExpress LLC. (Shanghai, China). We also used 6-bromohexyl (triphenyl) phosphanium (CAS No. 83152-22-1) Jskchem Ltd. (Shanghai, China). The other chemical reagents, including K_2_CO_3_, CH_2_Cl_2_, and KBr, were also purchased from Aladdin Ltd. (Shanghai, China). CH_2_Cl_2_ was distilled in the presence of CaH_2_, and THF was distilled with sodium/benzophenone under nitrogen protection. All chemicals were of commercial analytical reagent grade.

#### 2.1.2. Synthesis of (4-(6-(Triphenylphosphonio)hexyl)oxy)phenyl)-3-methoxyphenyl)-4,4,5,5-tetramethyl-4,5-dihydro Imidazole Radical (TPP-NIT)

**Scheme 1:**
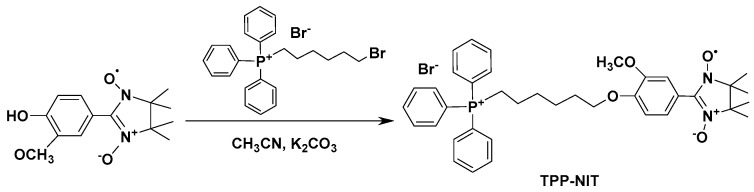
The synthesis route for TPP-NIT.

The synthetic route for the synthesis of the (4-(6-(triphenylphosphonio)hexyl)oxy)phenyl)-3-methoxyphenyl)-4,4,5,5-tetramethyl-4,5-dihydro imidazole radical (TPP-NIT) is shown in Scheme 1 (the complete synthesis method is shown in Supplementary Scheme S1). A total of 0.199 g (0.8 mmol) of the (4-hydroxy-3-methoxyphenyl)-4,4,5,5-tetramethyl-4,5-dihydro imidazole radical (see Supplementary Figures S1 and S2), 0.28 g (1.0 mmol) of 6-bromohexyl(triphenyl) phosphanium, and 0.138 g K_2_CO_3_ (1.0 mmol) were all added to 30 mL of acetonitrile and stirred at 60 °C. The reaction process was monitored via thin-layer chromatography (TLC). After reacting for approximately 8 h, the mixture was infiltrated to remove the precipitate, and a crude blue liquid was obtained. Using column chromatography, the crude blue liquid was purified (dichloromethane:methanol = 12:1) to obtain a dark blue oil (TPP-NIT) with a yield of 72.5%. IR(KBr): ν 1357, 1265 (N-O), 1114 (C-O) cm^−1^ (see Supplementary Figure S3). HRMS (ESI): *m*/*z* [M-Br]^+^ calcd for C_28_H_28_ClN_4_O_2_: 624.3111, found: 624.3101 (see Supplementary Figure S4). 

#### 2.1.3. Preparation of Nanoparticles (NPs-TPP-NIT)

The preparation of nanoparticles is shown in Figure 1C. TPP-NIT was encapsulated in PLGA using a modified emulsion solvent evaporation method. In brief, the organic phase was composed of PLGA (10 mg/mL; 50:50; CAS number: 34346-01-5, Sigma Aldrich, St Louis, MO, USA), TPP-NIT (1 mg/mL, synthesized by our laboratory, supporting information) and dichloromethane (DCM). The water phase included sodium cholate (SC; 1 mg/mL; CAS number: 361-09-1, TCI, Shanghai, China) and D-α-tocopherol polyethylene glycol succinate (TPGS; 3 mg/mL; CAS number: 9002-96-4, Sigma Aldrich, St Louis, MO, USA). The organic phase and aqueous phase of the above composition were mixed and emulsified in an ice bath for 90 s. Then, the mixture was stirred using a magnetic stirrer for 5 h to allow the solvent to continuously evaporate, thereby removing organic solvents and obtaining nanodispersions. Then, the nanodispersion was added to centrifugal filters with a molecular weight limit of 10 kD and collected via centrifugation. Finally, the nanosuspension was redispersed in double-distilled water (ddH_2_O) to obtain the nanoparticles (NPs-TPP-NIT).

#### 2.1.4. Characterization of NPs-TPP-NIT

Using a Nano ZSP Zetasizer (Malvern Instruments, Malvern, UK), dynamic light scattering (DLS) was carried out to measure the particle size, polydispersity index (PDI), and ζ electric potential at room temperature. The NP surface morphology was characterized using a scanning electron microscope (SEM—Quattro S; Thermo Fisher, Brno-Černovice, Czechia). The NP encapsulation efficiency (EE) was measured using a high-performance liquid chromatography system (Agilent Technologies, Inc., Santa Clara, CA, USA) with a reverse-phase C18 column (F90104; 4.6 × 250 mm; Capcell Pak MG, Beijing, China). The NP encapsulation efficiency (EE) and drug loading were measured using a high-performance liquid chromatography system (Agilent Technologies, Inc., Santa Clara, CA, USA) with a reverse-phase C18 column (F90104; 4.6 × 250 mm; Capcell Pak MG, Beijing, China) in triplicate. The hemolytic activity of NPs was measured on a spectrophotometer (WD-2102B; Beijing Liuyi Biotechnology, Beijing, China). To clarify the biological distribution of NPs, the nanoparticles labeled with phthalocyanine 5.5 (Cy5.5; HY-D0924; MedChem Express, Monmouth Junction, NJ, USA) were prepared using the method used for the PLGA-encapsulated TPP-NIT, except TPP-NIT was replaced with Cy5.5. 

### 2.2. Cells

The human normal hepatocytes cell line (L-02), human liver cancer cells (HEPG2), human gastric epithelial cells (GES), human moderately well-differentiated gastric cancer cells (MKN28), and human poorly differentiated gastric cancer cells (MKN45) were provided by the Department of Chemistry, the Air Force Medical University (No. 169, Changle West Road, Xi’an, Shaanxi 710032, China). The cells were cultured in Dulbecco’s modified Eagle’s medium (DMEM) at 37 °C and a humidity of 5% CO_2_ supplemented with 10% fetal bovine serum, 50 μg/mL streptomycin, and 10,000 units/mL penicillin. 

### 2.3. Animals

BALB/c male mice (weight, 18–22 g; SPF grade) were provided by the Center for Experimental Animals at the Fourth Military Medical University. The mice were kept in a constant environment. The temperature was maintained at 23 ± 2 °C, the relative humidity was 60 ± 5%, and the light conditions consisted of a 12 h light/12 h dark cycle. The mice were allowed free access to tap water and pellet food. The number of animals used and the pain experienced by the animals was minimized. Animal experiments were approved by the Animal Care and Use Committee of the AFMU (Certificate No. KY20194116) following the Guidelines for the Care and Use of Laboratory Animals in China. 

### 2.4. Cell Uptake and Distribution In Vivo of NPs-TPP-NIT

L-02 cells were inoculated into a 6 cm culture dish and cultured for 24 h. Then, 1 mg/mL Cy5.5 labeled nanoparticles were dissolved in 10 mL of 1640 complete culture medium (serum-free) and the L-02 cells were incubated with the above nanoparticle suspension for 12 and 24 h. The cells were digested with trypsin after incubation. Then, the cells were inoculated into a 20 mm confocal culture dish. After the cells adhered to the wall and grew for 12 h, they were washed with PBS 4 times, and the culture medium was removed. Afterwards, PBS was absorbed and 480 mL of DiD working solution (C1995s, Beyotime, Haimen, China) was added and incubated at 37 °C for 15 min. The working solution was removed and the cells were deeply washed 4 times with PBS. Then, cells were fixed using a 4% paraformaldehyde fixative for 15 min. After washing with PBS three times, 250 μL of DAPI was added and incubated for 15 min. The DAPI (DAPI; C10012, Beyotime, Haimen, China) was then removed and fixed with paraformaldehyde for 10 min for observation under an Olympus DP80 fluorescence microscope. Sample images were observed at excitation wavelengths of 644 nm and emission wavelengths of 655 nm.

The Cy5.5-labeled NPs (10 mg/kg^−1^ in 200 μL PBS) were administered to male BALB/c mice (body weight, 18–22 g; specific-pathogen-free (SPF) grade) intravenously (i.v.). Then, at 0, 1, 6, 12, 24, and 48 h, primary organs, including the spleens, livers, lungs, and kidneys, were harvested from the sacrificed mice. We used a Kodak in vivo imaging system to image Cy5.5 fluorescence in these organs (IVIS Spectrum; PerkinElmer Corp., Waltham, MA, USA).

### 2.5. Cytotoxicity Assay

The cell counting kit 8 (CCK-8) method was conducted to perform the cytotoxicity testing. L-02 cells were cultured in DMEM/F12 containing a 10% fetal bovine serum until the logarithmic growth phase was reached; then, they were digested by 0.25% trypsin. Cells were inoculated into a 96-well plate (1 × 10^4^ cells/well). Then, the cells were cultured under suitable conditions for 24 h at 37 °C in a humidified atmosphere with 5% CO_2_. Amifostine (WR2721) or NPs-TPP-NIT were dissolved in 1640 complete culture medium at the final concentrations of 1.5 × 10^−8^, 1.5 × 10^−7^, 1.5 × 10^−6^, 1.5 × 10^−5^, 1.5 × 10^−4^, 1.5 × 10^−3^, and 1.5 × 10^−2^ mM. The cells were cultivated with these solutions at 100 μL per well in a 96-well plate for 24 h. Each concentration had 5 duplicate wells in one plate. 

### 2.6. Protective Effects of NPs-TPP-NIT on L-02 Cells after X-ray Irradiation

The protective effect of NPs-TPP-NIT on radiation damage in L-02 cells was measured using the CCK-8 method. The L-02 single-cell suspension was diluted to a cell concentration of 5000 cells/mL using a complete culture medium containing 20% fetal bovine serum 1640; then, 100 μL of the suspension per well was added to the cells and the cells were cultured for 24 h in a humidified atmosphere with 5% CO_2_ at 37 °C. Afterward, the culture medium was removed and 100 μL of the NP-TPP-NIT solution at different concentrations (1.5 × 10^−8^, 1.5 × 10^−7^, 1.5 × 10^−6^, 1.5 × 10^−5^, 1.5 × 10^−4^, and 1.5 × 10^−3^ mM) was added, and the concentration of WR2721 was 1.5 × 10^−3^ mM. After incubation for 2 h, the cells were irradiated with 2 Gy, 4 Gy, 6 Gy, and 8 Gy X-rays, respectively. The dosage rate of the X-ray irradiation was approximately 1.414 Gy·min^−1^. The cells continued to be cultured for 72 h after irradiation. Then, the cells were incubated with 100 µL of 10% CCK-8 for 1 h. Using PBS as a control, the optical density (OD) at 570 nm was determined using a spectrophotometer. Five replicates were used for each concentration. In addition, the influence of NPs-TPP-NIT on the cell viability of GES, HEPG2, MKN28, and MKN45 cells after 6 Gy X-ray irradiation was estimated using the CCK-8 method. The pretreatment method of NPs-TPP-NIT (0.15 µM) and WR2721 (1.5 µM) on these cells was the same as the L-02 cell culture method mentioned above. After 2 h of treatment, the cells were exposed to a dose of 6 Gy X-ray radiation. Then, the cell viability was determined using the method mentioned. Five replicates were used for each concentration.

### 2.7. Cell Apoptosis

In 6-well plates, L-02 cells at a concentration of 5 × 10^3^ cells per well were allowed to attach for 24 h. Before being exposed to 6 Gy X-ray irradiation (1.414 Gy·min^−1^), the cells were treated with a 0.15 µM NP-TPP-NIT solution for 1 h. After 0.5 h of X-ray irradiation, the cells were washed with PBS three times and then incubated in DMEM culture for 24 h. Then, the apoptosis rate of L-02 cells was determined by a FACS flow cytometer, using the annexin V-fluorescein isothiocyanate (annexin V-FITC) apoptosis detection assay kit (Beyotime Biotech Inc., Shanghai, China).

### 2.8. Western Blot Analysis

We used the BCA assay kit to determine the protein concentrations and separated proteins of total extracts on 10% SDS gels (W003-1-1, Nanjing Jiancheng Bioengineering Institute, Nanjing, China). Separated proteins were electrophoretically transferred onto polyvinylidene difluoride membrane (PVDF) (WGPVDF45, Servicebio, Wuhan, China) and blocked with 5% non-fat milk in phosphate-buffered saline (PBS)-Tween for 2 h. The primary antibodies were polyclonal rabbit anti-Bcl-2 (1:1000, GB124830, Servicebio, China), anti-BAX (1:1000, GB12690, Servicebio, China), anti-cleaved-caspase 3 (1:1000, GB11767C, Servicebio, China) and β-actin (1:2000, GB11001, Servicebio, China). They were incubated with the membranes at 4 °C overnight. The membranes were incubated with a secondary antibody conjugated to horseradish peroxidase (Beyotime, Shanghai, China) after being washed with TBST three times for 10 min each time. Bands were visualized using an ECL system (G2014-50ML, Servicebio, China). The ChemiDoc-It2 610 imaging system (UVP, LLC, Upland, CA, USA) was used to conduct the visualization, and Image-Pro Plus 6.0 (Media Cybernetics, Inc., Bethesda, MD, USA) was used for quantification. All experiments were independently replicated three times.

### 2.9. In Vivo Toxicity Assay of NPs-TPP-NIT

The BALB/c male mice were assigned to 4 groups (6 mice in each group): (i) control group, (ii) 7-day NP-TPP-NIT, (iii) 14-day control, and (iv) 14-day NP-TPP-NIT. The mice in groups (ii) and (iv) were treated with NPs-TPP-NIT (5 mg/kg) through intraperitoneal injection once a day, for either 7 days or 14 days consecutively. After 7 or 14 days of administration, blood samples from each group of mice were collected for blood biochemical examination, and major organs including the heart, liver, spleen, lungs, and kidneys were collected for histopathological examination. 

(1) Hematology and blood biochemistry examinations. Approximately 100 µL of blood was collected through the eye socket and was maintained at 4 °C for 4 h. The blood sample was centrifuged at 1500 rpm for 10 min and tested for alanine aminotransferase (ALT), aspartate aminotransferase, total protein (TP), blood urea nitrogen (BUN), and albumin (ALB). 

(2) Histopathology examinations. The main organs of the mice were fixed with formaldehyde (4%) and processed into paraffin blocks. After staining with hematoxylin and eosin (H&E), the slides were imaged using a fluorescence inverted microscope.

### 2.10. Survival Assays In Vivo

Male mice were randomly divided into a normal group, an IR exposure group, and an NP-TPP-NIT (5 mg/kg) pre-treatment group (*n* = 10). A total of 30 min prior to irradiation exposure, the drug solution was administered to mice via intravenous injection. The mice in the normal group and the IR group were given an equal amount of distilled water. Thirty minutes after administration, all mice except for those in the normal group received a single dose of 6-Gy X-ray total body irradiation at the Radiation Center of AFMU, with an irradiation duration of 8 min and a dose rate of 1.414 Gy·min^−1^. The overall health status, including fur condition, behavior, food intake, and bowel movements, was measured. The survival rates of mice in different groups were observed over 30 days. In addition, the protective effect of NPs-TPP-NIT on the main organs of mice irradiated with 6 Gy X-ray was also observed on the 7th day. The organs of the 6 Gy X-ray group and the 6 Gy X-ray + NP-TPP-NIT group were fixed with 10% (*v*/*v*) buffered formalin for 24 h, dehydrated in ethanol, and embedded in paraffin. Next, the organs were sliced into 5 µm thick sections, stained with hematoxylin and eosin (H&E), and observed under an Olympus BX41 microscope (Olympus, Tokyo, Japan). The hematological examination experiment was conducted as follows: on the 7th and 14th days after irradiation, approximately 100 µL of the blood from the eyes of mice from different groups (*n* = 6) was collected into anticoagulant tubes (potassium EDTA) for hematological examinations to estimate the effect of NPs-TPP-NIT on the level of WBCs, RBCs and PLTs of irradiated mice.

### 2.11. Statistical Analysis

The significant differences between the groups were determined by analyzing data using SPSS version 21.0 (IBM Corp., Armonk, NY, USA). The mean standard deviation was used to represent the experimental results. In addition, one-way analysis of variance was also tested, as well as the Tukey multiple comparison test using the least significant difference (LSD) test. In all cases, *p* < 0.05 indicates a significant difference.

## 3. Results

### 3.1. Preparation and Characterization of NPs-TPP-NIT

To obtain nanoparticles with higher drug loading and an appropriate particle size, a simple and reproducible emulsion solvent evaporation technique was applied to prepare the nanoparticles. The preparation process of NPs-TPP-NIT is presented in Figure 1C. The results for particle size, PDI, and ζ potential of NPs-TPP-NIT are shown in Figure 1D,E. The hydrodynamic diameter of NPs-TPP-NIT is concentrated around 88.62 ± 2.75 nm, their PDI is 0.248 ± 0.02, and their negative ζ potential is −38.3 ± 3.24 mV, meaning that the nanosystems are stable. Figure 1F shows the SEM image of the nanoparticles, demonstrating that the NP is spherical, has a smooth surface, and remains dispersed without aggregation. It is generally believed that NPs < 200 nm can improve blood retention characteristics based on enhanced permeability and retention (EPR) effects, avoiding rapid liver clearance and increased accumulation at tumor sites. The PDI of NPs-TPP-NIT ranged from 0.1 to 0.3, which indicated that the distribution of NPs is uniform and their size distribution is narrow. In addition, NPs-TPP-NIT showed good drug EE, up to 95.05 ± 1.83%, as well as less hemolysis (<3%) compared to the positive control (Figure 1G). Therefore, NPs-TPP-NIT could be administered intravascularly within certain concentration limits.

### 3.2. Cell Uptake and Distribution of NPs-TPP-NIT In Vivo

**Figure 2:**
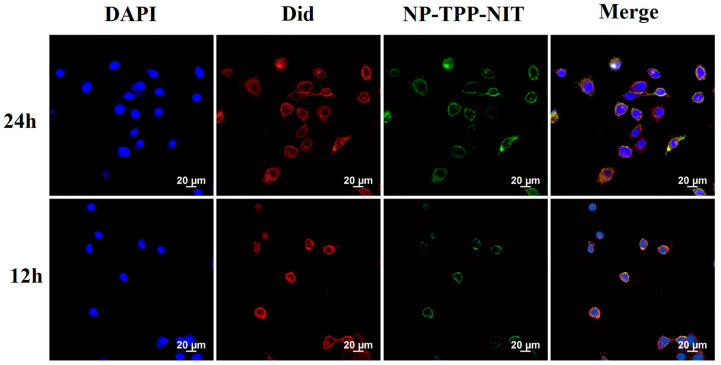
The cellular uptake rate of NPs-TPP-NIT in L-02 cells. L-02 cells were incubated with 1 mg/mL cy5.5-labeled nanoparticle suspension for 12 and 24 h. The maximum excitation wavelength of DAPI is 364 nm, the maximum emission wavelength is 454 nm, the maximum excitation wavelength of Did was 644 nm, and the maximum emission wavelength is 665 nm (Scale bar: 20 µM, *n* = 5).

The uptake of nanoparticles by L-02 cells was observed using a laser confocal microscope, as shown in Figure 2. After 24 h of incubation with Cy5.5-labeled nanoparticles, a large amount of green fluorescence was distributed inside the cells, almost all of which was taken up by the cells. The cellular uptake of nanoparticles has a certain time-dependent effect.

**Figure 3:**
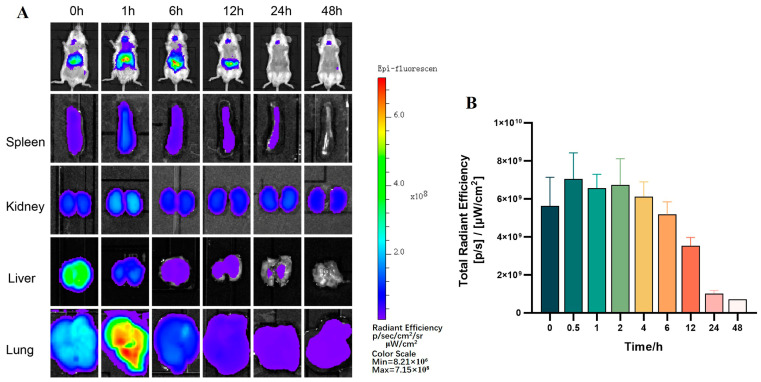
Biodistribution of NP-TPP-NIT in vivo. (**A**) Typical ex vivo images and (**B**) quantitative results of the average fluorescence intensity of NP-TPP-NIT (-Cy5.5) in the livers, spleens, lungs, and kidneys at 0, 1, 6, 24, and 48 h after administration. The color bar represents the average radiance efficiency (ph s^−1^ cm^−2^ s r^−1^)/(μW cm^−2^). Data are mean ± standard deviation (*n* = 3).

Images were obtained and fluorescence quantification was determined in certain primary organs at different time points after NP-TPP-NIT administration to study the long-term biological distribution of NPs-TPP-NIT in mice (Figure 3A,B). The results indicate that NPs-TPP-NIT were rapidly distributed throughout these organs, within 2 h after administration. As NPs-TPP-NIT circulated, fluorescence signals in mouse spleens, livers, lungs, and kidneys gradually weakened, exhibiting time-dependent clearance. Forty-eight hours after administration, weak fluorescence intensity was still detectable in these organs, indicating the retention of NPs therein, which might be beneficial for prolonging the radioprotective period of the drug in these tissues.

### 3.3. Cytotoxicity and Protective Effects of NPs-TPP-NIT against Radiation Injury to L-02 Cells

**Figure 4:**
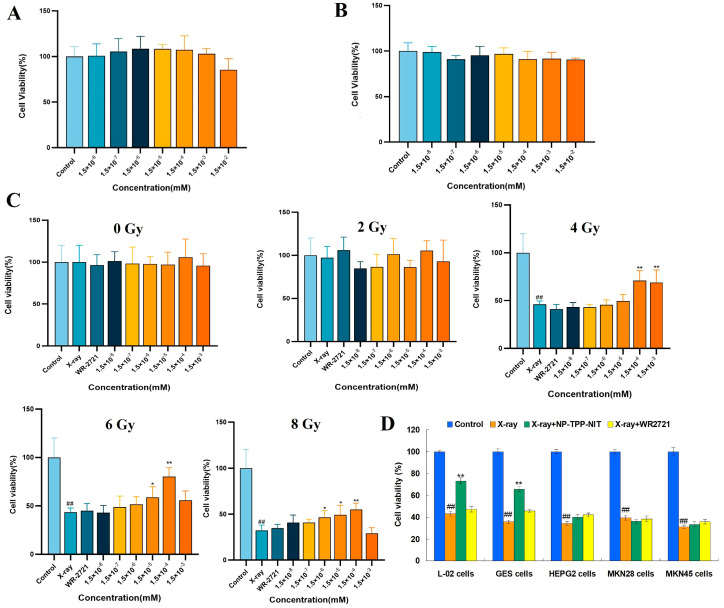
The cytotoxicity of different concentrations of NPs-TPP-NIT (**A**) and amifostine (**B**) on L-02 cells (*n* = 6). (**C**) The radioprotective effect of NPs-TPP-NIT and amifostine on the viability of L-02 cells exposed to 2, 4, 6, 8 Gy X-ray irradiation (1.414 Gy min^−1^). (**D**) The influence of NPs-TPP-NIT on cell viabilities. ## *p* < 0.01 vs. normal control group, * *p* < 0.05, ** *p* < 0.01 vs. X-ray group (*n* = 6).

When the concentration of NPs-TPP-NIT and WR2721 ranged from 1.5 × 10^−8^ to 1.5 × 10^−2^ mM, no significant inhibition effect on the cell viability of L-02 cells compared with the control group and no cytotoxicity were detected (Figure 4A,B). Therefore, the administration concentrations of NPs-TPP-NIT and WR2721 were selected within the non-toxic concentration range to study the protective effects they have on cells from damage induced by X-ray irradiation.

To study the anti-radiation effect of NPs-TPP-NIT in a cell culture, we examined the viability of irradiated L-02 cells using a CCK-8 assay. As is shown in Figure 4C, compared with the blank group, low-dose X-rays (2 Gy) caused less damage to the vitality of L-02 cells. When L-02 cells were irradiated with medium doses of 4 Gy and 6 Gy X-rays, the viability of L-02 cells was significantly inhibited. Moreover, the survival rate of the X-ray group (without any drugs added) was less than 50%. After 4 Gy X-ray irradiation, 0.15 μM and 1.5 μM nanoparticles significantly improved cell survival rates, reaching 71.6% and 68.0%, respectively. There was no statistically significant difference in the effect of these two nanoparticle concentrations on cell survival rates. The protective effect with 6 Gy X-ray irradiation was similar to that with 4 Gy X-ray irradiation, with a cell survival rate of 78.3% in the 0.15 μM nanoparticle group. After high-dose X-ray (8 Gy) radiation, the cell survival rate of the X-ray group further decreased to only 33.4%. In contrast, the 0.15 μM nanoparticles still showed strong activity in improving the survival rate of L-02 cells (cell survival rate 55.8%). The results indicate that the nanoparticles had significant protective effects against the damage of 4 Gy, 6 Gy, and 8 Gy X-rays to L-02 cells.

In addition, the influence of NPs-TPP-NIT on the cell viability of GES, HEPG2, MKN28, and MKN45 cells after 6 Gy X-ray irradiation was estimated. The results show that after 6 Gy X-ray radiation, there was no significant difference in the proliferation rate between the tumor cells pretreated with NPs compared to the untreated groups. This result indicates that the NPs-TPP-NIT we prepared do not influence the effect of radiation on tumor cells to inhibit proliferation (Figure 4D).

### 3.4. Antioxidant Damage Effect and Inhibition of Apoptosis of NPs-TPP-NIT

**Figure 5:**
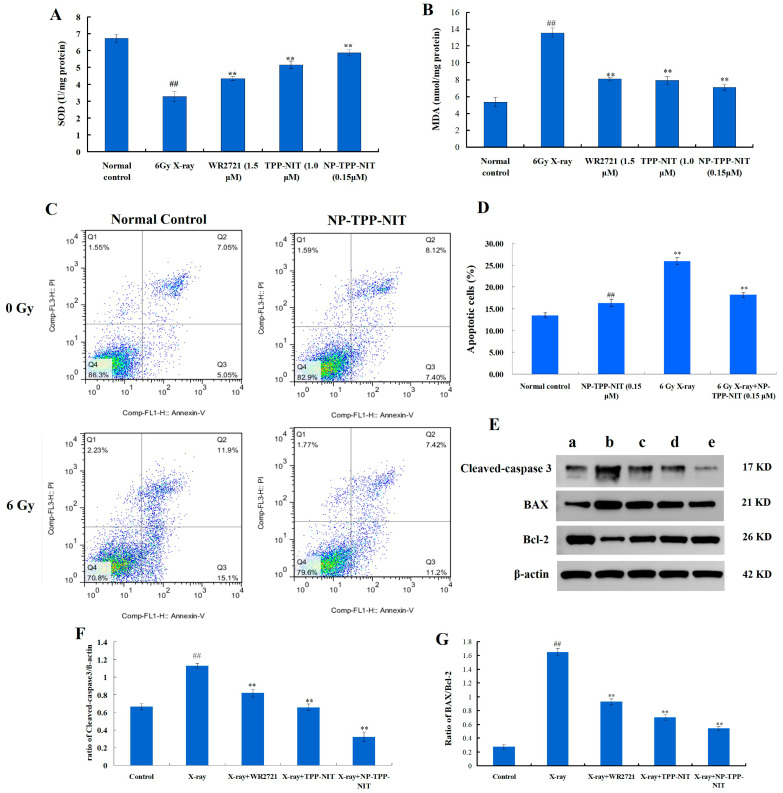
The effect of NPs-TPP-NIT on the level of SOD (**A**) and MDA (**B**) in L-02 cells (*n* = 6). (**C**) Representative images of the number of apoptotic cells recorded on a flow cytometer after cells were stained with annexin V/PI in the normal control group, 0.15 μM NP-TPP-NIT group, 6 Gy X-ray group, and 6 Gy X-ray + 0.15 μM NP-TPP-NIT group. Viable cells are in the lower left quadrant (Q4), early apoptotic cells are in the lower right quadrant (Q3), late apoptotic or necrotic cells are in the upper right quadrant (Q2), and necrotic cells are in the upper left quadrant (Q1). (**D**) Quantitative analysis of the percentage of apoptotic cells in the non-irradiation groups or the groups after 24 h of 6 Gy irradiation. Apoptotic cells were determined by counting the percentage of Annexin V-positive cells. Data are expressed as the mean ± S.D. (*n* = 3), ## *p* < 0.01 vs. normal control group, ** *p* < 0.01 vs. 6 Gy-X ray group. (**E**) Representative Western blots of Bcl-2, Bax, and cleaved-caspase 3 expression. a: Control group; b: 6 Gy X-ray group; c: 6 Gy X-ray + 1.5 μM WR2721 group; d: 6 Gy X-ray + 1.0 μM TPP-NIT group; e: 6 Gy X-ray + 0.15 μM NP-TPP-NIT. (**F**) Quantitative analysis of the ratio of cleaved-caspase 3/β-actin and (**G**) the ratio of Bax /Bcl-2. Data are expressed as the mean ± S.D., *n* = 3. ## *p* < 0.01 vs. control group, ** *p* < 0.01 vs. 6 Gy-X ray group. The uncropped blots are shown in Figure S5.

The level of superoxide dismutase (SOD) and malondialdehyde (MDA) in L-02 cells after X-ray irradiation are shown in Figure 5A,B. The results show that compared with the normal control group, X-ray irradiation significantly decreased SOD levels and significantly increased MDA activity (*p* < 0.01). However, NP-TPP-NIT pretreatment significantly reduced MDA activity in L-02 cells (*p* < 0.01) and increased SOD levels (*p* < 0.05). 

To investigate the impact of NPs-TPP-NIT on radiation-induced apoptosis, the population of apoptotic cells was analyzed with flow cytometry after annexin V and propidium iodide (PI) double-staining (Figure 5C). After 6 Gy irradiation, the percentage of apoptotic cells was increased from ~12.1% (control group) to ~27.0% (6 Gy X-ray group), while this significantly decreased to ~18.6% in the NP-TPP-NIT group (Figure 5D). In addition, NPs-TPP-NIT did not induce obvious apoptosis by themselves without radiation at the concentration of 0.15 μM. Furthermore, the expressions of Bax, Bcl-2, and cleaved-caspase 3 were also measured, and the results are shown in Figure 5E–G. The results show that the anti-apoptotic protein Bcl-2 was significantly downregulated compared with the control group in L-02 cells, while the pro-apoptotic proteins Bax were upregulated. In addition, the ratio of Bax/Bcl-2 increased significantly, induced by 6 Gy X-ray irradiation (*p* < 0.01). Moreover, the expression of cleaved-caspase 3 of the irradiated cells increased significantly compared to the normal L-02 cells. NPs-TPP-NIT significantly downregulated the ratio of Bax/Bcl-2 and decreased the expression of cleaved-caspase 3 compared with the X-ray group (*p* < 0.01). These results were consistent with those of staining, indicating that NPs-TPP-NIT strongly relieved radiation-induced cell apoptosis in L-02 cells.

### 3.5. In Vivo Toxicities of NPs-TPP-NIT

**Figure 6:**
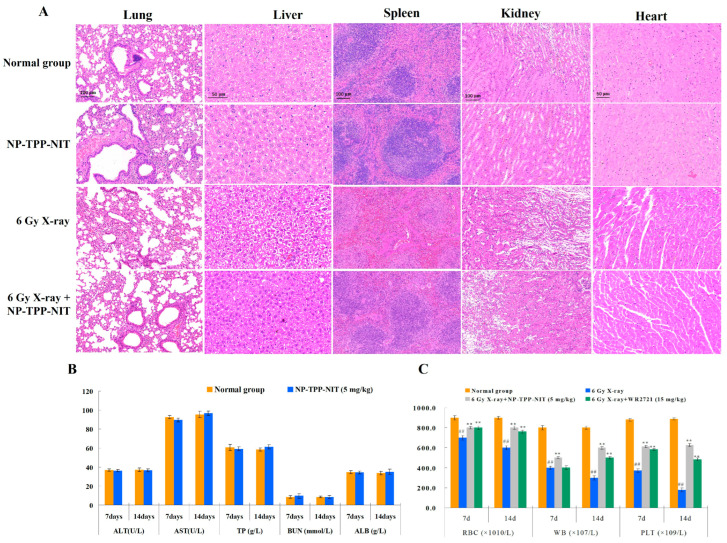
(**A**) Representative images of H&E staining of some of the main organs of the mice treated and not treated with NPs-TPP-NIT (5 mg/kg) or exposed to 6 Gy X-ray irradiation scale bars = 50 μM. (**B**) Effects of NPs-TPP-NIT (5 mg/kg) on the level of ALT, AST, TP, BUN and ALB in serum for 7 and 14 consecutive days. (**C**) Effects of NPs-TPP-NIT (5 mg/kg) on red blood cells (RBC), white blood cells (WBC) and platelets (PLT) in the peripheral blood of mice exposed to 6 Gy X-ray irradiation (1.414 Gy min^−1^) for 7 and 14 days. ## *p* < 0.01 vs. normal control group, ** *p* < 0.01 vs. X-ray group (*n* = 6).

The results of histopathological and blood biochemical examinations were used to evaluate the in vivo toxicity of NPs-TPP-NIT. The effect of NPs-TPP-NIT (5 mg/kg) on the pathological changes in some of the main organs on the 14th day was examined using an H&E staining assay. As is described in Figure 6A, no obvious damage was found in the major organs such as the heart, liver, spleen, lung, and kidney in mice at the experimental time point. Furthermore, there were no significant differences compared to the normal group. Meanwhile, we also studied the protective effects of NPs-TPP-NIT on the main organs of mice after radiation (Figure 6A). The results show that 6 Gy X-ray irradiation could lead to certain damage to these major organs, while NP-TPP-NIT pretreatment partially reverses this damage. For instance, the spleens of mice in the IR group were characterized by monocytic infiltration and splenic follicular atrophy. NP-TPP-NIT treatment maintained the normal morphology of the splenic follicles.

As is shown in Figure 6B, it can be seen that there was no significant difference in ALT, AST, ALB, and TP between the mice treated with NPs-TPP-NIT for 7 or 14 days and the untreated mice. Although the mice in the NP-TPP-NIT group showed an increase in BUN on day 7 compared to the control group, their BUN returned to normal levels after 14 days. Therefore, NPs-TPP-NIT have no significant side effects on the liver and kidneys of mice. Figure 6C shows the changes in the number of RBCs and WBCs as well as the content of PLTs in the peripheral blood of mice within 14 days after 6.0 Gy of X-ray irradiation. As is shown in Figure 6C, the number of RBCs, WBCs and PLT in the peripheral blood of mice after irradiation showed a downward trend after 14 days. The results show that NPs-TPP-NIT had a significant inhibitory effect on the reduction in the number of RBCs, WBCs, and PLTs caused by irradiation compared to the 6 Gy X-ray group (*p* < 0.01).

### 3.6. Effect of NPs-TPP-NIT on the Survival Rate of Mice after Irradiation Exposure

**Figure 7:**
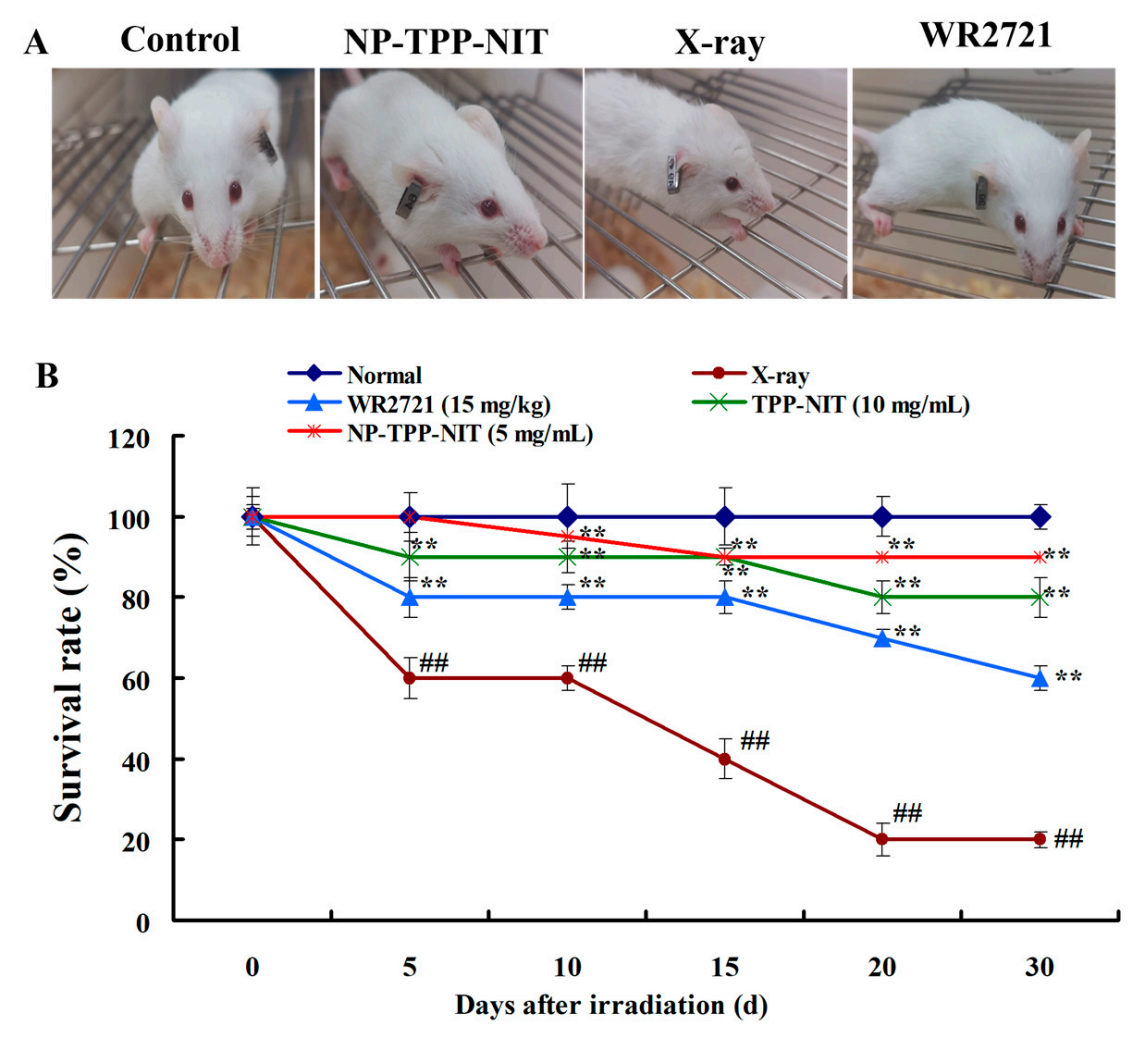
The effects of NPs-TPP-NIT on the survival rate of mice exposed to 6.0 Gy X-ray radiation. (**A**) Mouse images and (**B**) survival rate of mice. The data are expressed as the percentage of surviving mice. Data are presented as the mean ± S.D., *n* = 10. ** *p* < 0.01 compared with the X-ray radiation exposure group; ## *p* < 0.01 compared with the normal control group.

The survival state of the mice in each group was observed and recorded, as shown in Figure 7A. The skin and hair of mice in the IR group dried up, their movements were slow, their spirits were depressed, and their appetites decreased after irradiation exposure. In the NP-TPP-NIT-treated groups, the above physical signs of the mice were improved. The effects of NPs-TPP-NIT on the survival rate of the mice after irradiation exposure are shown in Figure 7. The results demonstrate that when the exposure dose of X-ray irradiation was 6.0 Gy, the survival rate in the IR group was 60% on the 10th day, 20% on the 20th day, and 20% on the 30th day after exposure (Figure 7B). This was significantly lower than that in the normal group (*p* < 0.05). NPs-TPP-NIT (5 mg/kg) can achieve a survival rate of 70% in mice on the 30th day. Compared with the IR group, there was a significant increase in the survival rate (*p* < 0.05), indicating that NPs-TPP-NIT have a strong protective effect on irradiated mice.

## 4. Discussion

Radiotherapy is one of the traditional and promising treatments for patients with cancer, but it may also cause severe side effects [3,4]. Exposure to IR is known to result in organ injury. Radiotherapy using ionizing radiation is becoming increasingly widespread, and the associated side effects experienced by organisms have also become a problem worth studying. The development of radioprotective agents has been a focal point for a long time. Direct or indirect irradiation (through the radiolysis of water-producing reactive oxygen species, ROS) interacts with macromolecules (such as DNA, lipids, and proteins) causing oxidative damage to tissues, and finally, organ dysfunction, through a series of biochemical and physical disturbances [20]. This disturbance is observed in terms of the increased production of ROS leading to lipid peroxidation, the oxidation of DNA and proteins, as well as the activation of pro-inflammatory factors that subsequently lead to the occurrence of diseases [21,22]. Thus, the search for radioprotectors to mitigate radiation damage is necessary and will be of extensive use in radiotherapy [23].

**Figure 8:**
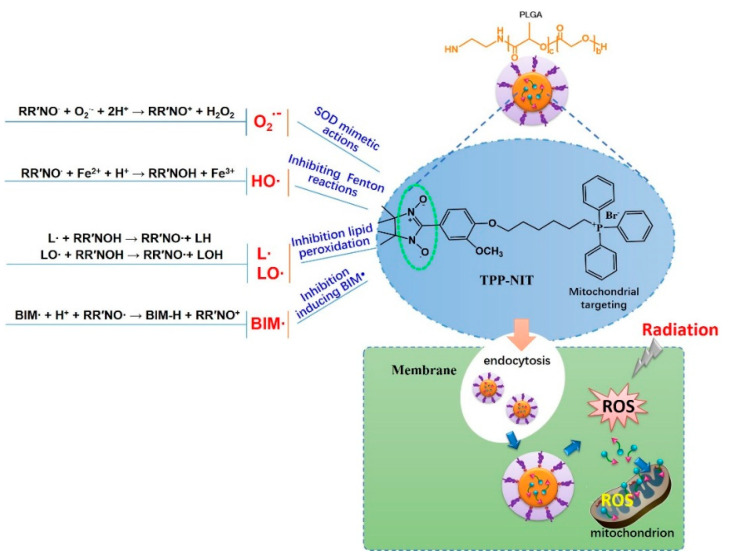
NPs-TPP-NIT can play a protective role by participating in some endogenous redox reactions to reduce the deleterious effects of radiation.

To date, the FDA has approved some radioprotective drugs for clinical use, such as amifostine (WR2721), filgrastim, pegfilgrastim, and sargramostim [24]. WR2721 is an organic thiophosphate that can be metabolized to generate compounds containing sulfhydryl groups. The thiol metabolites of WR2721 can clear ROS, which allows WR2721 to selectively protect normal tissues from acute and late radiation damage, but it does not have a protective effect on tumor tissues [25]. However, the side effects of WR2721 seriously limit its clinical use [26]. Imidazole nitronyl nitroxide radicals (NITs, Figure 1A) are new and efficient scavengers that have been developed in recent years. They have been shown to reduce oxidative damage in some experimental models. This is due to their imidazole ring nitrogen atom and the oxygen atom bonded to it, which can receive or contribute one to two electrons, causing them to transition between three different chemical structures: nitrogen oxide radicals, reduced hydroxylamines, and oxidized ammonium oxide cations (Figure 8). NIT has a similar free-radical scavenging effect to endogenous SOD, and it is not consumed during the decomposition of O_2_ˉ^·^ into O_2_ and H_2_O_2_. It is also known as a catalytic free-radical scavenger [27]. NITs can react with the membrane lipid free radicals L∙ and LO∙ to protect the cell membrane, mitochondrial membrane, and other biomembranes [28]. NIT can also prevent Fenton reactions via accepting electrons from reducing metal complexes, thereby inhibiting the generation of ·OH radicals.

The nano-delivery systems developed in the past decade have obvious advantages, such as significantly improving the stability of drugs in the body, prolonging the cycle and metabolism time of drugs, increasing the sustained action time of drugs against damage, and reducing the dosage of drugs used. For example, Nguyen et al. prepared a stable nanomelanin and found that X-rays only caused mild splenic fibrosis in 40% of nanomelanin-protected mice, whereas severe fibrosis was observed in 100% of mice treated with X-rays alone [29]. The nanomelanin also partly rescued the volume and weight of mouse spleens from irradiation by promoting the transcription levels of splenic interleukin 2 (IL-2) and tumor necrosis factor alpha (TNF-α). In this study, we successfully loaded a TPP-NIT onto PLGA and obtained nanoscale particleswith the size range of 80–200 nm (Figure 1D). This increased the effective distribution in the absorption of radiation, and provided excellent antioxidant activity for nitronyl nitroxide radicals to scavenge ROS generated during and after X-ray irradiation. These characteristics of NPs-TPP-NIT may have contributed to the partial protection of mouse spleens from X-ray irradiation attack. 

Although our research demonstrated that NPs-TPP-NIT have a protective effect in reducing IR-induced damage, there are still some limitations. For example, the relationships between the protective effect of NPs-TPP-NIT and the IR-induced redox imbalance and the oxidative damage and apoptosis signaling pathways remain to be explored. Furthermore, more in vivo tumor models need to verify the ability of NPs-TPP-NIT to confer protection against radiation in addition to the safety of radiotherapy treatment.

## 5. Conclusions

In this study, we prepared and characterized NPs-TPP-NIT and investigated its ability to protect cells and healthy tissues of mice from the attack of X-rays during radiation. Our results demonstrated that NPs-TPP-NIT could effectively protect the organs of mice from X-ray irradiation with an antioxidant effect. Further, our results revealed that treatment with NPs-TPP-NIT strongly promoted overall survival in X-ray-irradiated mice. Taken together, we suggest that NPs-TPP-NIT are promising radioprotectors and, therefore, could be applied in radiotherapy to protect healthy tissues and organs from radiation during the treatment of cancer.

## Data Availability

Data are contained within the article and Supplementary Materials.

## Supplementary Materials

The following supporting information can be downloaded at: https://www.mdpi.com/article/10.3390/cancers16020351/s1. Scheme S1. The synthesis method for preparation of TPP-NIT. Figure S1. The IR spectrum of 4-hydroxy-3-methoxybenzaldehyde nitroxyl radical. Figure S2. The HRMS spectrum of 4-hydroxy-3-methoxybenzaldehyde nitroxyl radical. Figure S3. The IR spectrum of TPP-NIT. Figure S4. The HRMS spectrum of TPP-NIT. Figure S5. a, Control group; b, 6 Gy X-ray group; c, 6 Gy X-ray + 1.5 μM WR2721 group; d, 6 Gy X-ray + 1.0 μM TPP-NIT group; e, 6 Gy X-ray + 0.15 μM NPs-TPP-NIT.

## Abbreviations

IR: ionizing radiation; SOD: superoxide dismutase; MDA: malondialdehyde; ROS: reactive oxygen species; WR2721: sulfhydryl compound amifostine; NITs: nitronyl nitroxyl radicals; H&E: hematoxylin and eosin; PLGA: poly(lactic-co-glycolic-acid); SEM: scanning electron microscopy; TEM: transmission electron microscope.
