# Supplementary material for: Mitochondria-Targeted Nitronyl Nitroxide Radical Nanoparticles for Protection against Radiation-Induced Damage with Antioxidant Effects
Source: Cancers (Basel). 2024 Jan 13;16(2):351. doi: 10.3390/cancers16020351 (PMC10814804; doi:10.3390/cancers16020351)
Supplement: Supplementary file 1 [file cancers-16-00351-s001.zip › cancers-2817926-supplementary.pdf]

Electronic Supporting Information

Mitochondrial-targeted nitronyl nitroxide radicalnanoparticles for protection against radiation-induced damage with antioxidant effect

Shigao Huang [1,2*,†], Min Xu [3,†], Qingyue Da [4], Linlin Jing [4] and Haibo Wang [3,*]

[1] Department of Cell Biology, National Translational Science Center for Molecular Medicine, The Air Force Medical University, Xi'an 710032, China

[2] Department of Radiation Oncology, Xijing Hospital, The Air Force Medical University, Xi'an 710032, China

[3] Department of Chemistry, School of Pharmacy, The Air Force Medical University, Xi'an 710032, China

[4] Centre for Translational Medicine, The First Affiliated Hospital, Xi'an Jiaotong University, Xi'an 710061, China; daqy21@lzu.edu.cn (Q.D.); jinglinlin@xjtufh.edu.cn (L.J.)

* Correspondence: huangshigao2010@aliyun.com (S.H.); haibo7691@fmmu.edu.cn (H.W.)

† These authors contributed equally to this work.

1. Chemical Materials

2,3-Bis(hydroxylamino)-2,3-dimethylbutane was synthesized in our laboratory[1]. The chemical reagents were purchased from Aladdin Ltd. (Shanghai, China). CH_2Cl_2 was distilled in the presence of CaH_2, and THF was distilled with sodium/benzophenone under nitrogen protection All chemicals were of commercial analytical reagent grade.

2. Synthetic methods

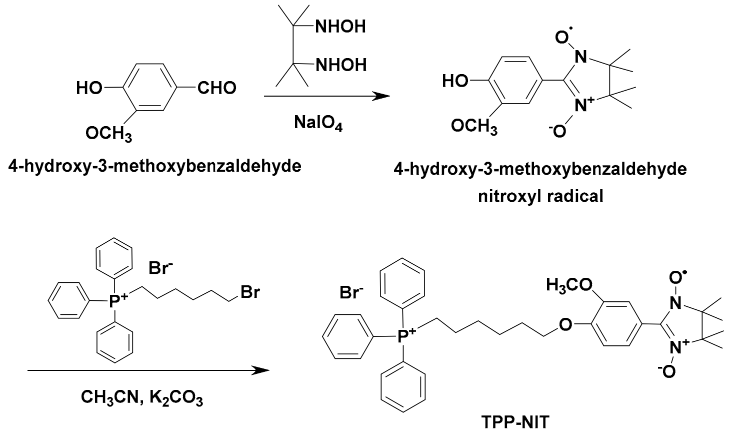

Scheme S1. The synthesis method for preparation of TPP-NIT.

2.1 Procedure for the preparation of (4-hydroxy-3-methoxyphenyl)-4,4,5,5-tetramethyl-4,5-dihydro imidazole radical (scheme 1). A solution of 4-hydroxy-3-methoxybenzaldehyde (0.76 g, 5 mmol) and 2,3-bis (hydroxylamino)-2,3-dimethylbutane (0.74 g, 5 mmol) in methanol (20 ml) was stirred at room temperature for 12 h. After the end of the reaction, the solvent was removed and the residue was suspended in 200 ml CH_2Cl_2. The saturated aqueous solution of $NaIO_4$ was dropwised slowly to the mixture solution and stirred at 0°C until the reaction solution appears dark blue. The organic phase was separated, and the aqueous phase was extracted with CH_2Cl_2 (20 mL×3). The combined organic layers were dried over anhydrous Na_2SO_4. The deep blue solution was then evaporated. The crude product was purified by column chromatography on silica gel using absolute ethyl acetate/*n*-hexane (1:3) as eluent, giving a deep blue solid product (1.16 g, 83.1 %). m.p. 96.5-98.1°C. IR(KBr): ν 3355 (OH), 1388, 1353 (N-O), 1215, 1122 (C-O-C) cm^{-1}. HRMS (ESI): m/*z* $[M+Na]^+$ calcd for $C_{28}H_{28}ClN_4O_2$: 302.1243, Found: 302.1250.

2.2 **Synthesis of (4-(6-(triphenylphosphonio)hexyl)oxy)phenyl)-3-methoxyphenyl)-4,4,5,5-tetramethyl-4,5-dihydro imidazole radical (TPP-NIT).** The synthetic route for the synthesis of TPP-NIT was shown in Scheme 1. 0.199 g (0.8 mmol) of (4-hydroxy-3-methoxyphenyl)-4,4,5,5-tetramethyl-4,5-dihydro imidazole radical, 0.28 g (1.0 mmol) of 6-bromohexylphosphine bromide and 0.138 g (1.0 mmol) of K_2CO_3 were dissolved in 30 ml acetonitrile. The mixture was stirred and reacted at 60°C for 8 h. Thin layer chromatography (TLC) was used to monitor the reaction process. When the reaction was complete, the solution

was filitrated and the solvent was removed under vacuum toobtain a crude blue liquid.Then the crude blue liquid was purifiedby column chromatography (the eluent was dichloromethane: methanol = 12:1) to obtain dark blue oil. Yield 72.5%. IR(KBr): ν 1357, 1265 (N-O), 1114 (C-O) cm^{-1}. HRMS (ESI): m/*z* $[M-Br]^+$ calcd for $C_{28}H_{28}ClN_4O_2$: 624.3111, Found: 624.3101.

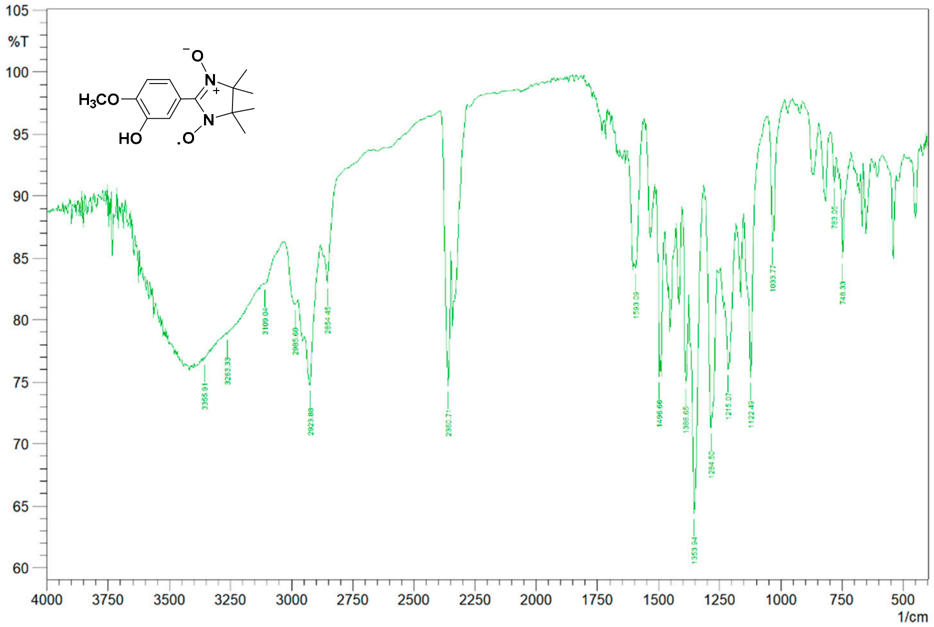

Figure S1. The IR spectrum of 4-hydroxy-3-methoxybenzaldehyde nitroxyl radical.

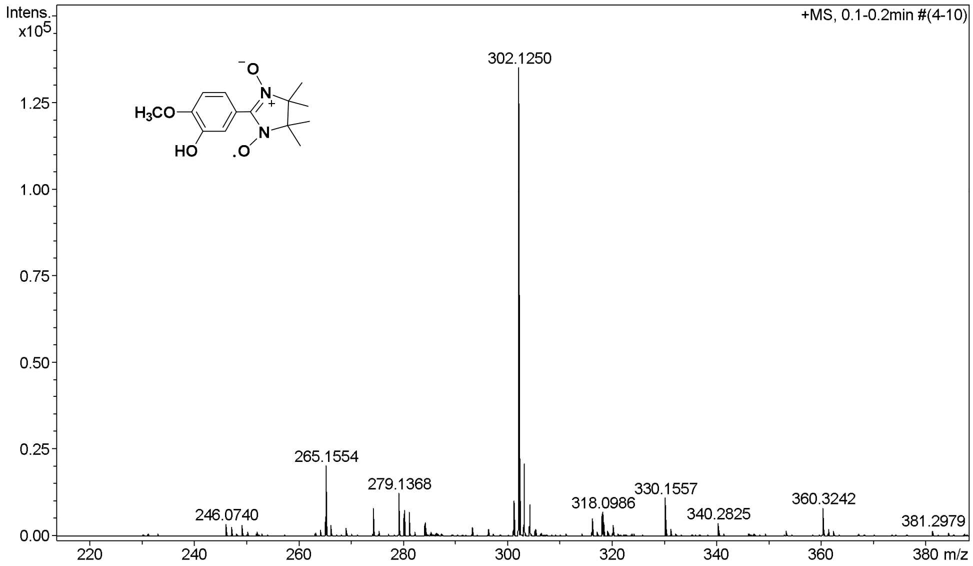

Figure S2. The HRMS spectrum of 4-hydroxy-3-methoxybenzaldehyde nitroxyl radical.

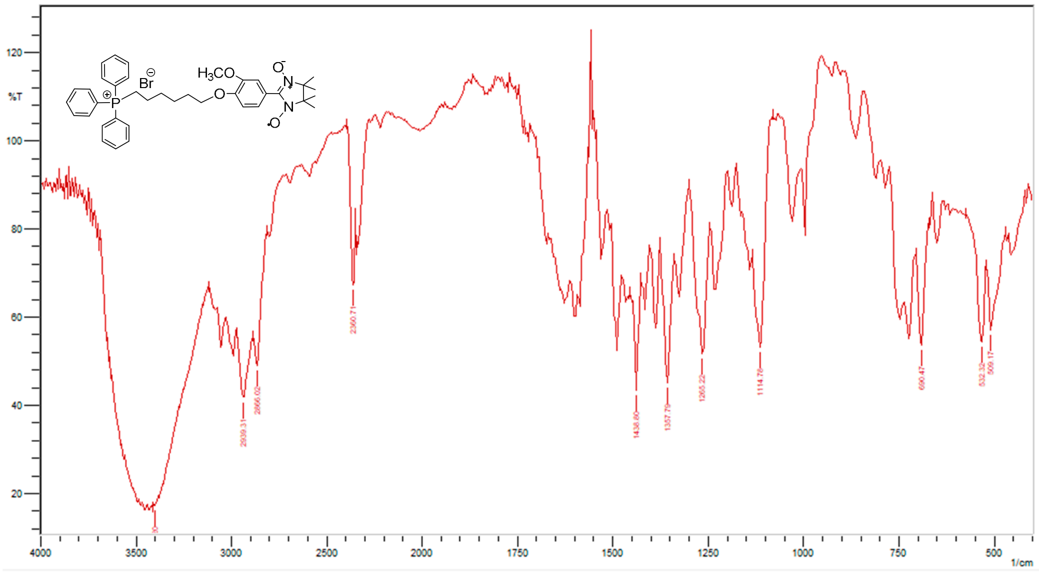

Figure S3. The IR spectrum of TPP-NIT.

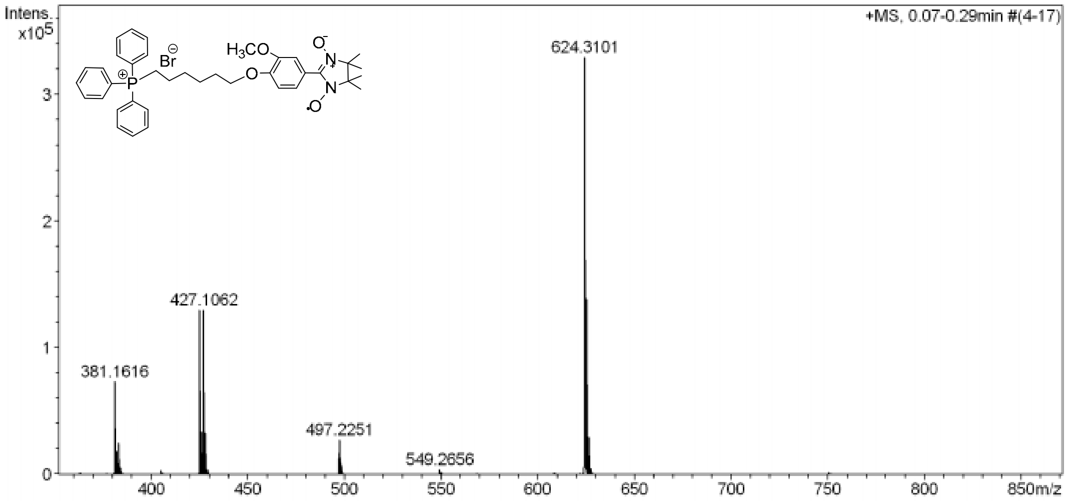

Figure S4. The HRMS spectrum of TPP-NIT.

3. Western Blot Analysis

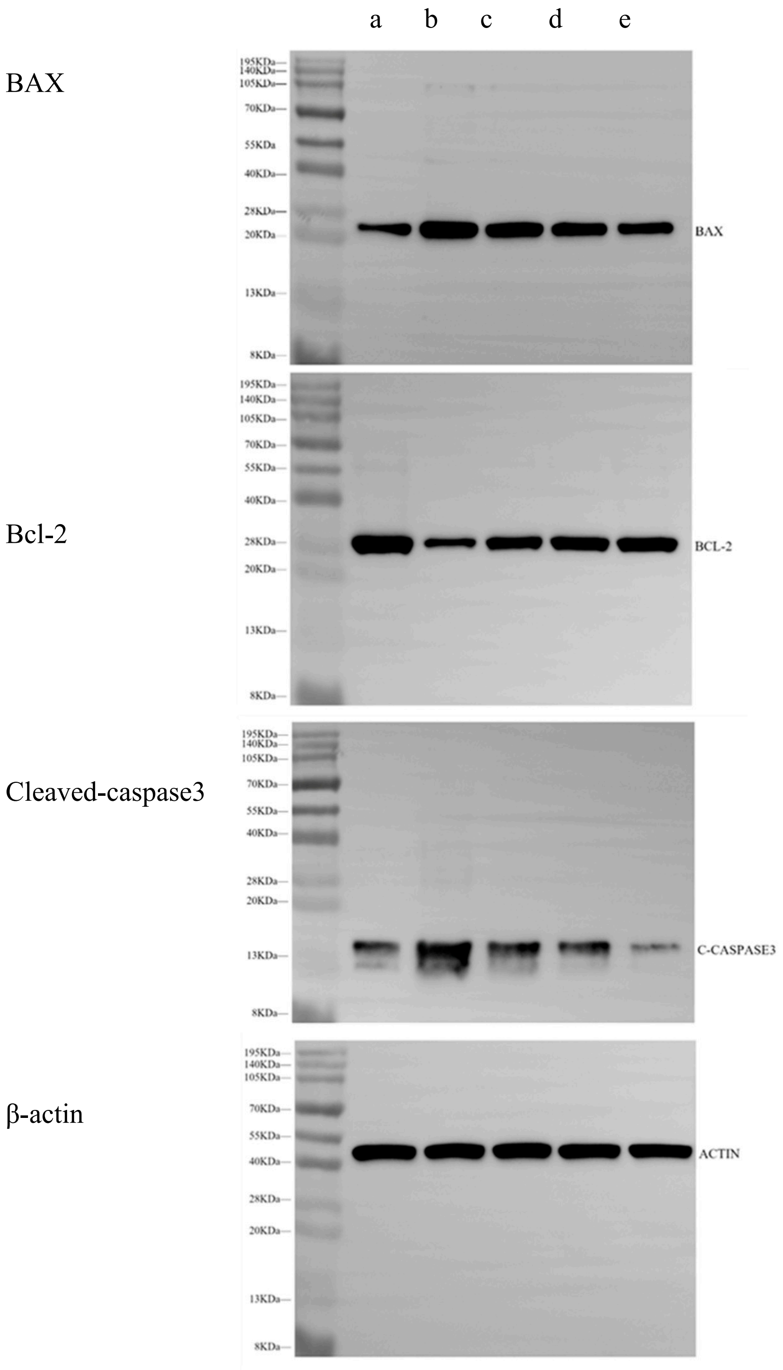

Figure S5. a, Control group; b, 6 Gy X-ray group; c, 6 Gy X-ray + 1.5 μM WR2721 group; d, 6 Gy X-ray + 1.0 μM TPP-NIT group; e, 6 Gy X-ray+0.15 μM NPs-TPP-NIT

References

[1] Haibo Wang, Yujing Jia, Peng Gao, Ying Cheng, Ming Cheng, Chentao Lu, Siyuan Zhou, Xiaoli Sun. Synthesis, radioprotective activity and pharmacokinetics characteristic of a new stable nitronyl nitroxyl radical-NIT2011. Biochimie 95 (2013) 1574-1581.
